# Predictors associated with successful weaning of veno-venous extracorporeal membrane oxygenation and mortality in adult patients with severe acute lung failure: Protocol of a pooled data analysis of cohort studies

**DOI:** 10.1371/journal.pone.0303282

**Published:** 2024-05-17

**Authors:** Yaxin Ning, Linya He, Keqi Pan, Weiwen Zhang, Jian Luo, Yan Chen, Zubing Mei, Danqiong Wang

**Affiliations:** 1 The Second School of Clinical Medicine, Zhejiang Chinese Medical University, Hangzhou, China; 2 School of Medicine, Shaoxing University, Shaoxing, Zhejiang, China; 3 Department of Critical Care Medicine, Quzhou People’s Hospital, The Quzhou Affiliated Hospital of Wenzhou Medical University, Quzhou, China; 4 Department of Anorectal Surgery, Anorectal Disease Institute of Shuguang Hospital, Shuguang Hospital, Shanghai University of Traditional Chinese Medicine, Shanghai, China; 5 Anorectal Disease Institute of Shuguang Hospital, Shanghai, China; UN Mehta Institute of Cardiology and Research Center, INDIA

## Abstract

**Background:**

Severe acute lung failure (ALF) often necessitates veno-venous extracorporeal membrane oxygenation (VV-ECMO), where identifying predictors of weaning success and mortality remains crucial yet challenging. The study aims to identify predictors of weaning success and mortality in adults undergoing VV-ECMO for severe ALF, a gap in current clinical knowledge.

**Methods and analysis:**

PubMed, EMBASE, and the Cochrane Central Register of Controlled Trials will be searched for cohort studies examining the predictive factors of successful weaning and mortality in adult patients on VV-ECMO due to severe ALF. Risk of bias assessment will be conducted using the Newcastle-Ottawa scale for each included study. The primary outcomes will be successful weaning from VV-ECMO and all-cause mortality. Between-study heterogeneity will be evaluated using the I^2^ statistic. Sensitivity, subgroup, and meta-regression analyses will be performed to ascertain potential sources of heterogeneity and assess the robustness of our results. We will use the Grading of Recommendations, Assessment, Development, and Evaluations (GRADE) tool to recommend the level of evidence.

**Discussion:**

This study seeks to provide clinically significant insights into predictors for weaning and mortality during VV-ECMO treatment for ALF, aiming to support clinical decisions and potentially influence health policy, thereby improving patient outcomes.

**Ethics and dissemination:**

Given the absence of direct engagement with human subjects or access to personal medical records, ethical approval for this study is deemed unnecessary. The study findings will be shared at a scientific conference either at the global or national level. Alternatively, the results will be presented for publication in a rigorously peer-reviewed journal regarding critical care medicine.

## Introduction

Acute Respiratory Distress Syndrome (ARDS), severe pneumonia, and other etiologies of acute severe lung failure can deteriorate rapidly, leading to the onset of hypoxic or hypercapnic respiratory failure in adult patients. Despite significant medical advancements, the prognosis remains poor with high mortality rates [1–4]. When conventional mechanical ventilation fails to provide adequate gas exchange, veno-venous extracorporeal membrane oxygenation (VV ECMO) serves as a valuable alternative [5, 6].

VV-ECMO has seen increased usage in recent years, particularly in managing cases of acute lung failure refractory to other rescue therapies. It functions by providing extracorporeal life support, thus allowing the lungs to rest and repair [7–10]. Despite this life-saving potential, the decision to implement ECMO is not without challenges, often owing to its high resource requirement, critical care infrastructural demands, and associated complications [11–13].

Successful weaning off VV-ECMO and improving survival are paramount goals, and identifying predictors to these ends is crucial to optimize patient management and resource allocation [14, 15]. Several patient and treatment factors such as the patient’s age, comorbidities, cause of acute lung failure, duration of ECMO, among others, have been suggested as potential predictors, but with varying consistency across differing studies [16–18].

Given the high disease burden and the vital role ECMO plays, a consolidated, systematic evaluation of these predictors is essential. An improved understanding of the factors associated with ECMO outcomes can guide clinical decision-making, provide centerpiece information for discussions with patients and their families regarding realistic goals and expectations, and offer directions for future research [19, 20].

Despite the noteworthy increase in high-quality cohort studies within the last decade, a consensus regarding these predictors remains elusive. Although the definitive validation of these factors would ideally be derived from randomized controlled trials, ethical and logistical obstacles often constrain their implementation in critically ill settings. Therefore, observational studies significantly contribute to our understanding and provide indispensable insights regarding these predictors. This study details a forthcoming pooled analysis of high-quality cohort studies to identify predictors associated with successful weaning from VV-ECMO and mortality in adult patients with severe acute lung failure. This will be an invaluable addition to the body of evidence surrounding VV-ECMO and its decision-making process, contributing to more optimal patient management and improved survival outcomes.

## Methods

### Study registration

This study protocol has been registered in the OSF database (Open Science Framework) prior to implementation with the registration DOI of https://doi.org/10.17605/OSF.IO/PXF86. OSF (https://osf.io/) provides services for the registration of study protocols, ensuring transparency and decreasing the likelihood of duplicate work. Any updates or amendments to this initial protocol will be appropriately documented and dated on the register site in accordance with its guidelines. This protocol is designed following the principles of the Preferred Reporting Items for Systematic Review and Meta-Analysis Protocols (PRISMA-P) [21] and the study will be conducted according to Cochrane Handbook [22] to ensure comprehensive and consistent reporting of all the study components (S1 Table).

### Literature searches

A comprehensive literature search strategy will be developed to identify all pertinent studies. Electronic databases, including PubMed, EMBASE, and the Cochrane Library, will be systematically searched. The search strategy will encompass both subject headings (such as MeSH in PubMed and EMTREE in Embase) and free-text words to ensure a broad coverage. Search terms will include: "veno-venous ECMO", "VV ECMO", "lung failure", "successful weaning", "mortality", and related phrases (Table 1).

**Table 1:** Search strategy for electronic database (Pubmed).

No.	Search terms
	**Extracorporeal Membrane Oxygenation terms**
1	"Extracorporeal Membrane Oxygenation"[Mesh]
2	"Oxygenators, Membrane"[Mesh]
3	"Extracorporeal Circulation"[Mesh]
4	(ECMO or ECCO2R or ECCO2-R or ECCO-2-R or ECCO2RD or ECCO2RT or ECPR or ECLS or VAECMO or VVECMO or VVECLS or mECMO)[Title/Abstract]
5	membran* oxygenat*[Title/Abstract]
6	((veno-venous or venovenous or veno-arterial or venoarterial or arterio-venous or VV or VA) and (extracorp* or extra-corp*)) [Title/Abstract]
7	((extracorp* or extra-corp*) and (oxygen* or membran*))[Title/Abstract]
8	((extracorp* or extra-corp*) and (cardiopulmonary resuscitation or CPR or lung* or circulat* or blood gas)) [Title/Abstract]
9	(extrapulmonary oxygenation)[Title/Abstract]
10	((extracorp* or extra-corp*) and (CO2 or "CO(2)" or carbon dioxide)) [Title/Abstract]
11	((extracorp* or extra-corp*) and (gas transfer or gas flow or gas exchange or oxygen transfer or O2 transfer)) [Title/Abstract]
12	(Respiratory dialysis or carbon dioxide dialysis or ((CO2 or "CO(2)") and dialysis)) [Title/Abstract]
13	(lung assist*)[Title/Abstract]
14	((extracorp* or extra-corp*) and respiratory assist*)[Title/Abstract]
15	((extracorp* or extra-corp*) and (life support or lung support or respiratory support)) [Title/Abstract]
16	1-15/OR
	**Ventilator Weaning terms**
17	"Ventilator Weaning"[Mesh]
18	"Ventilators, Mechanical"[Mesh]
19	"Ventilators, Negative-Pressure"[Mesh]
20	"Respiration, Artificial"[Mesh]
21	"Positive-Pressure Respiration"[Mesh]
22	"Ventilator Weaning"[Mesh]
23	((mechanical* and ventilat*) or (ventilat* and (wean* or liberat* or extubat*)))[Title/Abstract]
24	"Mortality"[Mesh]
25	Survival[Mesh]
26	"Death"[Mesh]
27	(Mortality or survival or death or outcome*)[Title/Abstract]
28	17-27/OR
	**Acut lung failure terms**
29	"Respiratory Distress Syndrome"[Mesh]
30	(((acute or adult) and (respiratory adj1 distress)) or ards) [Title/Abstract]
31	"Acute Lung Injury"[Mesh]
32	((acute and lung* and injur*) or (shock and lung*)) [Title/Abstract]
33	29-32/OR
	**Cohort terms**
34	"Retrospective Studies"[Mesh]
35	"Cohort Studies"[Mesh]
36	"Longitudinal Studies"[Mesh]
37	"Follow-Up Studies"[Mesh]
38	"Prospective Studies"[Mesh]
39	"Registries"[Mesh]
40	(cohort or longitudinal or followup or prospective* or retrospective* or database* or population* or follow up or registry or registries or incidence OR prevalence OR mortality OR outcome OR progression OR natural history OR prognos* OR course* OR predict* OR population based OR epidemiologic OR case control) [Title/Abstract]
41	34-40/OR
	**Combined Terms**
42	16 AND 28 AND 33 AND 41

Existing systematic reviews on the topic will also be scrutinized for relevant studies. The references of included studies will be manually checked to identify any eligible study not captured by the database searches. Furthermore, relevant conference proceedings and clinical trial registries, particularly those focusing on critical care medicine, will be consulted to detect more recently conducted studies and grey literature.

The search will have no restrictions with respect to publication status (published and unpublished studies) or language. For non-English language publications, translations will be sought where possible. The search period will extend until December 2023 to ensure the capture of the most recent studies.

Two independent evaluators will assess the articles for eligibility based on pre-set inclusion and exclusion criteria. Disagreements between evaluators will be resolved through discussion, or by engaging a third evaluator if consensus cannot be achieved. Grey literature will also be explored to include study protocols and conference proceedings to avoid publication bias.

### Eligibility criteria and study selection

In accordance with the PICOS (Participants, Intervention, Comparator, Outcomes, Study design) framework for defining eligibility criteria, we have outlined the following criteria:

#### Population

Adult patients (age ≥18 years) who have been placed on VV-ECMO due to severe acute lung failure. Studies including pediatric populations or patients requiring veno-arterial ECMO for cardiac diseases will be excluded.

#### Intervention

The intervention is the utilization of VV-ECMO as a bridge to weaning for respiratory support in the context of severe ALF. Patients who are transitioned from VV-ECMO to a hybrid ECMO (such as veno-venous arterial or veno-arterial venous ECMO) mode during their treatment will be excluded.

#### Comparator

Comparators will be matched on key characteristics such as age, severity of illness, and ALF etiology. Studies without comparators will at least discuss standard care protocols for patients not receiving VV-ECMO.

#### Outcomes

Successfully weaned from VV-ECMO and mortality are the primary outcomes of interest. Eligible studies should provide quantifiable data on at least one of these primary outcomes in relation to a predictor.

#### Study design

Prospective and retrospective cohort studies will be incorporated. Excluded designs will be case reports, case series, cross-sectional studies, animal studies, reviews, letters, commentaries, and editorials.

Following a detailed search in the specified databases, the identified studies will be collected within a reference management software where duplicates will be removed. Independent screening of titles and abstracts retrieved will be performed by two evaluators according to the predetermined inclusion/exclusion criteria. Those fulfilling the criteria will undergo full text review. Disparities in evaluators’ choices will be addressed through discussions; a third evaluator will be involved if a consensus is not reached.

### Data extraction

We will adopt a standardized data extraction format to capture relevant information from the included studies. Two independent evaluators will extract the following data from each included study: first author’s name, year of publication, study design, country where the study was conducted, total sample size, patient characteristics (i.e., age, sex, underlying diseases), specific details about the ECMO procedure (e.g., duration of ECMO, indications for ECMO), primary and secondary outcomes, predictors under investigation, statistical methods employed, and key findings.

Information related to predictors for successful weaning and mortality will be systematically collated, together with the associated risk estimates like odds ratios (ORs), hazard ratios (HRs), and their 95% Confidential Intervals (CIs). If necessary, authors of the original studies may be contacted for further clarification or for additional unpublished data required for the analysis. Any inconsistencies between evaluators during the data extraction will be resolved through consensus or by involving a third evaluator.

### Risk of bias assessment

The Newcastle-Ottawa Quality Assessment Scale (NOS) will be used for assessing risk of bias in cohort studies, which evaluates studies on three broad categories including the selection of the study groups, the comparability of the groups, and the ascertainment of the output of interest for cohort studies [23]. Guided by the NOS, each study will be analytically assessed in these categories to determine the likelihood of bias. A maximum of nine stars can be awarded, with higher scores indicating superior quality and lower risk of bias. Studies with a score of six or less will be considered to have a high risk of bias. Two independent evaluators will conduct the assessment. Any disagreements will be resolved by consensus or third-party arbitration if consensus cannot be reached.

### Grading level of evidence

In an attempt to ascertain the quality of evidence for the selected cohort studies, we aim to adopt the Grading of Recommendations, Assessment, Development, and Evaluations (GRADE) approach [24], which provides a systematic and transparent framework crucial in grading the quality of evidence, offering comprehensive and tangible results to effectively aid in clinical decision-making.

In the GRADE system, preparedness begins with cohort studies being classified as ’low quality’. However, the quality level can be adjusted up or down depending upon several aspects, including:

1. Magnitude of effect (upgraded if statistically significant and clinically meaningful)
2. Presence of a dose-response gradient (upgraded)
3. All plausible residual confounding (upgraded if they would reduce a demonstrated effect or suggest a spurious effect if no effect was observed)
4. Study limitations or risk of bias (downgraded)
5. Consistency or unexplained heterogeneity (downgraded)
6. Precision (downgraded if wide confidence intervals)
7. Reporting bias (downgraded) [4].

Two independent evaluators will assess each cohort study using the GRADE methodology, evaluating the studies for potential pitfalls, such as risk of bias, indirectness, imprecision, inconsistency, and possibility of publication bias.

Discrepancies during the grading process will be resolved through discussion. If required, a third-party arbitrator will be enlisted. The results will be presented in the ’Summary of Findings’, using a ’GRADE Evidence Profile’ table.

### Outcome definition

The two primary outcomes for this protocol are the successful weaning off VV ECMO and overall mortality in the adult population experiencing severe acute lung failure. Successful weaning is defined as the ability to maintain oxygenation and ventilation without VV-ECMO support for at least 48 hours following decannulation. Mortality is described as all-cause patient death during hospitalization or within 90 days after decannulation [25].

### Statistical analysis

We will perform pooled data analyses using R software (‘meta’ and ‘metafor’ packages). In the primary analysis, we will analyze the pooled estimate of successful weaning off VV-ECMO in adults with severe acute lung failure. Meanwhile, in the secondary analysis, we will evaluate the pooled estimate of mortality rate in this adult patient population. To assess heterogeneity among the included studies, we will apply Cochrane’s Q statistic and the I^2^ statistic [26]. An I^2^ value of 0% implies no observed heterogeneity, while values greater than 50% indicate moderate to high heterogeneity. When significant heterogeneity is identified (I^2^ > 50%), a random-effects model will be utilized. If heterogeneity is low (I^2^ < 50%), a fixed-effects model will be used for analysis [27].

Meta-regression and subgroup analyses will also be undertaken to explore potential sources of heterogeneity and evaluate the impact of cohort-stable characteristics (such as patient demographics, clinical characteristics and study designs) on the pooled outcomes. Potential publication bias will be examined using Egger’s test, and a funnel plot will be created if more than 10 studies are included in the pooled data analysis [28]. In case of significant asymmetry indicating publication bias, the Duval and Tweedie’s Trim and Fill method will be implemented to adjust the results [29]. Finally, a sensitivity analysis will be performed to assess the robustness of the pooled data analysis results by sequentially excluding each study and reanalyzing the remaining ones. All statistical tests will be two-sided, and a p-value of less than 0.05 will be considered statistically significant.

### Amendments

During the review process, necessary updates or amendments will be implemented to this study protocol as required.

## Discussion

### Principal findings

Our study will shed light on crucial predictors impacting successful weaning from VV-ECMO and mortality among adult patients suffering from severe ALF. We hypothesize that multiple patient factors, as well as treatment regimens, may serve as predictors.

### Comparison with other studies

Previous studies have offered valuable insights into factors influencing successful VV ECMO weaning and related mortality [30]. Munshi et al. [6] carried out a meta-analysis addressing mortality and ECMO, revealing valuable predictors including the patient’s age and their health status before initiating ECMO. Yet, their focus wasn’t specifically on successful weaning. Similarly, Combes et al. [5] mainly directed their meta-analysis towards the benefit of ECMO versus conventional ventilation strategies for ARDS, with weaning success being secondary. Previous studies have analyzed the predictors individually but the overall evidence lacks integration and robust exploration. Our study will provide a significant contribution in this regard, by integrating evidence from multiple studies and providing more definite and clinically meaningful conclusions.

### Potential mechanisms

There are several potential mechanisms behind weaning success and mortality. The severity of lung failure, patient characteristics, associated comorbidities, and timeliness of ECMO initiation, among others, may critically contribute to these outcomes. Understanding the potential mechanisms underlying our findings will be an interpretive endeavour. If apparent predictors are subsequently identified, it will be essential to propose plausible pathophysiological pathways connecting these predictors to the outcomes, which can further facilitate future research and clinical applications. Also, if relevant, we will attempt to elaborate on the potential interplay of these predictors with established physiological markers and their potential role in pathogenesis, supporting the findings of other researchers in the field [31].

### Strengths and limitations

Among the many strengths of this study is the comprehensive approach we intend to take. The expansive time frame of our literature search, spanning over several decades, will allow us to capture a broad range of studies. The report of our study will strictly follow PRISMA guidelines [32], which can provide a sound methodological framework that ensures transparency, accuracy, and thoroughness in our analysis. The planned pooled data analysis will enable us to synthesize high-quality evidence from multiple studies, thereby potentially providing more precise estimates of predictors associated with the weaning success from VV-ECMO and mortality. This will further aid in building a comprehensive understanding necessary for guiding clinical decisions and can contribute substantially to the existing literature on ECMO in ALF. Another key strength is the prospective registration of our protocol, which reduces the risk of outcome reporting bias.

On the other hand, the study is also susceptible to several limitations. Foremost, the heterogeneity between studies could limit the comparability of the results. The differences might arise from various aspects such as in diagnostic criteria, approach to VV-ECMO, geographic variation, and management strategies in different settings. Further, definitions of ’successful weaning’ might differ across different studies.

## Conclusions and policy implications

Given the life-threatening nature of ALF and the important role of VV-ECMO as a life-support tool, our findings could have significant policy implications. If specific predictors tied to successful ECMO weaning are identified, they could serve in guidance and decision-making processes within critical care settings, potentially enhancing patient management. Our study could also draw attention to areas requiring further research and ideally pave the way towards standardizing ECMO weaning practices.

## Supporting information

S1 Table PRISMA-P checklist. (DOCX)
