# Correction: Predictors associated with successful weaning of veno-venous extracorporeal membrane oxygenation and mortality in adult patients with severe acute lung failure: Protocol of a pooled data analysis of cohort studies

**DOI:** 10.1371/journal.pone.0324188

**Published:** 2025-05-14

**Authors:** The PLOS One Staff

The affiliation for the eighth author is incorrect. Danqiong Wang is not affiliated with #2 but with #3: Department of Critical Care Medicine, Quzhou People’s Hospital, The Quzhou Affiliated Hospital of Wenzhou Medical University, Quzhou, China.

The publisher apologizes for the error.

## References

[1] Ning Y, He L, Pan K, Zhang W, Luo J, Chen Y, et al. Predictors associated with successful weaning of veno-venous extracorporeal membrane oxygenation and mortality in adult patients with severe acute lung failure: Protocol of a pooled data analysis of cohort studies. PLoS One. 2024;19(5): e0303282. doi: 10.1371/journal.pone.0303282 38758742 PMC11101029

